# Yoga for prenatal depression: a systematic review and meta-analysis

**DOI:** 10.1186/s12888-015-0393-1

**Published:** 2015-02-05

**Authors:** Hong Gong, Chenxu Ni, Xiaoliang Shen, Tengyun Wu, Chunlei Jiang

**Affiliations:** 1 Faculty of Psychology and Mental Health, Second Military Medical University, Shanghai, People's Republic of China; 2 Department of Pharmacy, Second Military Medical University, Shanghai, People's Republic of China

**Keywords:** Yoga, Prenatal depression, Systematic review, Meta-analysis

## Abstract

**Background:**

Prenatal depression can negatively affect the physical and mental health of both mother and fetus. The aim of this study was to determine the effectiveness of yoga as an intervention in the management of prenatal depression.

**Methods:**

A systematic review and meta-analysis of randomized controlled trials (RCTs) was conducted by searching PubMed, Embase, the Cochrane Library and PsycINFO from all retrieved articles describing such trials up to July 2014.

**Results:**

Six RCTs were identified in the systematic search. The sample consisted of 375 pregnant women, most of whom were between 20 and 40 years of age. The diagnoses of depression were determined by their scores on Structured Clinical Interview for DSM-IV and the Center for Epidemiological Studies Depression Scale. When compared with comparison groups (e.g., standard prenatal care, standard antenatal exercises, social support, etc.), the level of depression statistically significantly reduced in yoga groups (standardized mean difference [SMD], −0.59; 95% confidence interval [CI], −0.94 to −0.25; p = 0.0007). One subgroup analysis revealed that both the levels of depressive symptoms in prenatally depressed women (SMD, −0.46; CI, −0.90 to −0.03; p = 0.04) and non-depressed women (SMD, −0.87; CI, −1.22 to −0.52; p < 0.00001) were statistically significantly lower in yoga group than that in control group. There were two kinds of yoga: the physical-exercise-based yoga and integrated yoga, which, besides physical exercises, included pranayama, meditation or deep relaxation. Therefore, the other subgroup analysis was conducted to estimate effects of the two kinds of yoga on prenatal depression. The results showed that the level of depression was significantly decreased in the integrated yoga group (SMD, −0.79; CI, −1.07 to −0.51; p < 0.00001) but not significantly reduced in physical-exercise-based yoga group (SMD, −0.41; CI, −1.01 to −0.18; p = 0.17).

**Conclusions:**

Prenatal yoga intervention in pregnant women may be effective in partly reducing depressive symptoms.

**Electronic supplementary material:**

The online version of this article (doi:10.1186/s12888-015-0393-1) contains supplementary material, which is available to authorized users.

## Background

In Korea, 8%-12% of all pregnant women suffer with major depressive disorder (MDD), and about 20% have clinically significant depressive symptoms, which do not meet the criteria for MDD [1]. In the US, it is estimated that the prevalence of antenatal depression reaches 10–20% [2]. Indeed, prenatal depression is estimated to occur in 6–38% of pregnancies in different countries [3]. Prenatal depression, i.e., depressive episode during pregnancy, is undoubtedly a serious threat to the wellness of pregnant women all over the world. One study of 277 pregnant women indicated an antepartum depression rate of almost 20%, which was nearly double that of the 11% rate observed after delivery [4]. However, prenatal depression has been studied much less than postnatal depression [5]. Prenatal depression may negatively affect the physical and mental health of both mother and fetus [6]. For example, children of depressed mothers show lower birth weights, elevated resting heart rates, increased risk of developmental delays and prematurity, increased physiological reactivity, and more behavior problems in childhood and adolescence than children of non-depressed mothers [7-11]. Besides, prenatal depression has been regarded as the strongest risk factor for postnatal depression [12] and a mediator between risk factors and postnatal depression [13]. All of these highlight the necessity of prenatal interventions for depressed symptoms during pregnancy.

Antidepressant therapy can reduce symptoms of prenatal depression, but its safety has been controversially discussed for many years. Antidepressants may increase the risk of postpartum hemorrhage and harmfully affect the unborn child, therefore, they are not always safe during pregnancy [14,15]. Pregnancy is a major determinant of the cessation of antidepressant medication [16]. Only a small number of pregnant women with depressive disorders are using antidepressants because of the mixed data on fetal and neonatal outcomes [17]. Both psychotherapy and complementary and alternative medicine (CAM) are popular therapies. Psychological treatments for perinatal depression are extensively used, and have shown benefits in some studies [18]. CAM comprises a broad array of different treatments, ranging from herbal medicine to yoga. Many women are comfortable using CAM during pregnancy since these treatment options appear to be potentially useful for improving psychological health during pregnancy [19,20]. Most patients do believe that CAM treatments are safe and effective [21]. Notably, in Israel, most obstetricians show positive attitudes to these treatments and recommend using them during pregnancy [22].

With the goal of achieving the highest possible functional harmony between body and mind, the Indian yoga encompasses various domains, including ethical disciplines, physical postures and spiritual practices. All aspects of yoga practice contribute to a state of deep relaxation in which both the body and mind experience calmness [23]. In the United States, fifteen million adults have been practicing yoga, with almost half using yoga to prevent health problems, promote wellness or manage a specific health condition [24]. Recent studies indicate that yoga can improve life quality in physical conditions such as cancer [25], menopause [26] and pain [27]. Yoga intervention also plays a vital role in preventing mental disorders such as refractory epilepsy [28], schizophrenia [29], feelings of sadness [30], depression and anxiety disorders [23]. Moreover, the efficacies of yoga on pregnant women (e.g., reduce perceived stress, enhance immune function, improve adaptive autonomic response to stress, etc.) [31,32] and pregnancy outcome (e.g., gestational age at delivery, mode of delivery, intrauterine growth retardation, etc.) [33] have been identified. Especially, yoga has been shown to increase gestational age and birth weight [34], improve maternal comfort during labor [27], facilitate normal delivery, decrease complications, labor duration, and anesthesia requirements [27,33].

Here, we examined the evidence that yoga (exercise-based yoga and integrated yoga) may improve the depressive symptoms of pregnant women. This systematic review was carried out basing on several randomized controlled trials (RCTs) published up to July 2014.

## Methods

### Search strategy

An Internet-based search was performed through PubMed, Embase, the Cochrane Library and PsycINFO from all retrieved articles up to July 2014. For PubMed and Embase, the following search terms were used: the Medical Subject Heading (MeSH) or Emtree terms “depression”, “depressive disorder”, “mood disorder”, “prenatal depression”, “pregnancy”, “prenatal care”, “pregnancy complications” and “yoga” and the corresponding free terms. For the Cochrane Library and PsycINFO, the correlative keywords were used. Terms were moderately expanded within each electronic database whenever necessary. No language restrictions were applied.

### Inclusion and exclusion

Inclusion criteria included RCTs of pregnant women who were randomized to yoga and comparison groups. There were no strict restrictions on comparison groups. Therefore, any trials that compared yoga to usual care or any other physical or mental care (e.g., standard prenatal care, standard antenatal exercises, social support, etc.) were eligible. There are a number of different types of yoga being taught and practiced today. Some only included physical exercise, such as stretching, savasana, or other asana postures. In addition to physical exercise, other kinds of yoga, which were defined as integrated yoga, also included pranayama, meditation or deep relaxation. Therefore, both physical-exercise-based yoga and integrated yoga were accepted and evaluated in a subgroup analysis. The pregnant women who participated in the trials were either depressed or not depressed. Differences between the two types of pregnant participants were also investigated in the other subgroup analysis. Exclusion criteria included nonrandomized or uncontrolled trials, postpartum depression, still incomplete articles after contacting the authors, trials based on in vitro fertilization (IVF), treatment with medicine besides yoga, and antenatal depression pooled with other constructs. These selection criteria were confirmed according to the results of searching.

### Data extraction

Data extraction was completed by three authors (HG, CN and TW) using a standard extraction form. First, two authors independently extracted the data. Then, the third author compared their results and discussed with them to reach a consensus. Only those original articles, which not only fulfilled the inclusion criteria, but also did not meet the exclusion criteria, were regarded as qualified. The extraction form included the following information: publication year, country, the number of participants, the age and gestation of participants, the measurement of depression before and after intervention, cost, percentage of primiparous women in each group, exclusion criteria, the results of trials and the protocol of intervention group (the type of yoga, program length, frequency, duration, practice mode, etc.).

### Quality assessment

Two qualified reviewers (HG and CN) independently assessed the quality of the included studies. The quality assessment system was modified from the criteria of Juni and Stroup et al. [35,36], which included eligibility criteria, randomization, allocation concealment, lost to follow-up, intension-to-treat analysis and outcome assessor blinding. As this was an interventional study and it was quite difficult for the researchers to conduct the blinding of participant and provider, participant blinding and provider blinding were excluded in the score system. However, blinding of the outcome assessor could be adequate and it was included.

The quality of each study was assessed as “yes”, “unclear”, or “none”. The more criteria a study met the higher quality it had. Studies that met all the criteria were ranked as A. On the contrary, those could not meet any criteria were ranked as C. The remaining ones were ranked as B [37]. Another author (XS) participated in the discussion on the divided opinion, until an agreement was reached.

Since this study was a literature review of previously reported studies, ethical approval or additional consent from participants was not required.

### Data synthesis and statistical analysis

Most results of the trials only reported the pre-intervention and post-intervention means and standard deviations (SDs). However, the changes during the interventions were not reported. Although the trials were randomized, different baselines might still exist. Using the final mean and SD to analyze it was not accurate. According to the previous studies [38,39], a correlation coefficient of 0.6 was used to calculate the changing SD during the interventions. The calculation formula was based on the Cochrane handbook for systematic reviews of interventions [40].

Review Manager 5 software (version 5.3, The Nordic Cochrane Centre, Copenhagen, Denmark) for Windows package was used to analyze the data. For continuous outcomes, standardized mean differences (SMDs) with 95% confidence intervals (CIs) were calculated as the differences in means, and α = 0.05 was used as the statistical significant level. A fixed effects model was initially adopted to calculate χ^2^ and I^2^ to test for heterogeneity. It was regarded as notable heterogeneity, when I^2^ was valued at more than 50%. Since the random effects model is more conservative than the fixed effects model, a random effects model should be used unless it exhibited low heterogeneity [40,41]. Subgroup comparisons were also carried out to analyze the depression levels of different types of yoga and participants. The funnel plot, which is a scatter plot, has been used frequently to estimate the risk of publication bias. We did not use a funnel plot because of the limited number of included studies.

## Results

### Systematic review

**Figure 1:**
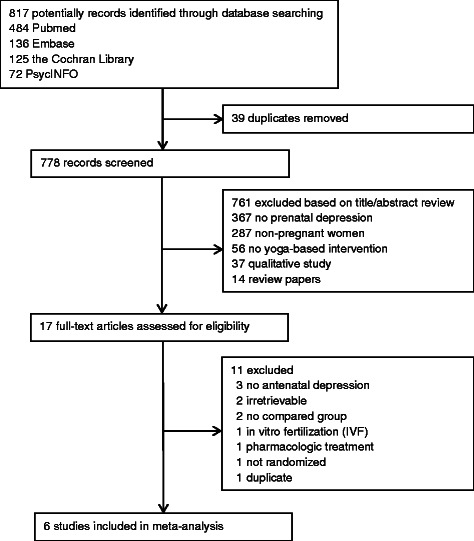
**Flow chart of study selection in meta-analysis.**

Of the 817 potentially relevant articles from the electronic database, only six were retained for analysis while the remaining 811 were excluded. Most were excluded because they were not relevant to prenatal depression (n = 367) or did not involve pregnant women (n = 287). For studies that were read in full text and assessed for eligibility, three were not about antenatal depression and were excluded [42-44], two were unable to obtain [45,46] (Figure 1), two reported without any controlled groups [47], one was in vitro fertilization (IVF) [48], one was treated with medicine [49], one did not indicate that randomization was conducted [50] and one [51] was already included in the present study [52]. Therefore, six RCTs (375 cases total) that compared the yoga groups with control groups for depression were finally included.

The characteristics of the six articles are shown in detail (please see Additional file 1: Table S1.) Most articles originated from the United States, while only one from India and another from United Kingdom. All of those six RCTs were published in the past five years. Four RCTs included depressed patients with a Structured Clinical Interview for DSM-IV (SCID) diagnosis of depression [53-56], while the other two trials enrolled normal or non-depressed pregnant women [52,57]. Three RCTs used exercise-based yoga [53,54,56], the other three RCTs used complex yoga interventions, including tai chi [55], relaxation, meditation [57], and breathing exercises [52]. Those RCTs mainly used Center for Epidemiological Studies Depression Scale (CES-D) [53-56], Hospital Anxiety Depression Scale (HADS) [57] and Edinburgh Postnatal Depression Scale (EPDS) [52] to measure the level of depression. Results of some articles showed significant differences favoring yoga over control groups [52-54,57], while others did not [55,56].

### Quality of included studies

Most included trials were not of high quality. All of them were randomly assigned to either a yoga treatment or a control group, but only two of them explained the randomization method [52,57]. Four RCTs reported the number of lost participants in each group and stated reasons for each case [52,55-57], but the remaining RCTs did not clarify this issue [53,54]. The allocation concealment and intension-to-treat analysis were not mentioned in most RCTs, except one [52]. As noted earlier, because it seemed impossible to adopt participant blinding and provider blinding in yoga intervention, only blinding of the outcome assessor was included. Adequate outcome assessor blinding was conducted in three RCTs [51,54,55]. All of the RCTs described the inclusion and exclusion criteria in detail. Three RCTs showed similar baseline [52,56,57], while the others did not [53-55]. The results of quality assessment are provided in this study (please see Additional file 2: Table S2).

### Meta-analysis

### Analysis of overall effect

**Figure 2:**
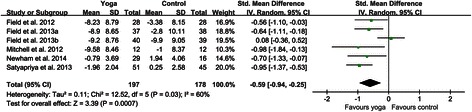
**Forest plots of effects of yoga on prenatal depression scores.** Forest plot of the comparison of the yoga intervention group versus the control group for prenatal depression scores.

Overall, six comparisons were made for depression. The results showed that there was a significant heterogeneity, as was evident from I^2^ = 60% (p = 0.03). When using a random effects model, the standardized weight mean difference (SMD) was −0.59 (95% CI, −0.94 to −0.25), which was a significant effect in favor of yoga (p = 0.0007) (Figure 2).

### Subgroup analyses

**Figure 3:**
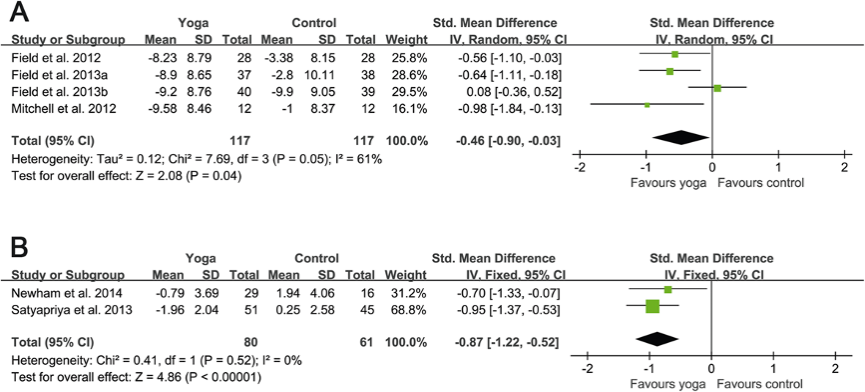
**Forest plots of effects of yoga on depression scores for prenatally depressed and non-depressed women. (A)** Forest plot of the comparison of the yoga intervention group versus the control group for depression scores in prenatally depressed women. **(B)** Forest plot of the comparison of the yoga intervention group versus the control group for depression scores in prenatally non-depressed women.

According to different types of participants and yoga interventions, two subgroups were created respectively. For different types of participants, four RCTs included pregnant women with depressive symptoms [53-56], while the remaining two RCTs included women without depressive symptoms [52,57]. Pooling trials according to the types of participants gave standardized mean difference of −0.46 (−0.90 to −0.03) for depressed women and −0.87 (−1.22 to −0.52) for non-depressed women (Figure 3). For the subgroup of depressed women, a random effects model was used, because there was a certain degree of heterogeneity within this subgroup (I^2^ = 61%, p = 0.05). While a fixed effects model was used in the subgroup of non-depressed women since there was no significant evidence of heterogeneity in this subgroup (I^2^ = 0, p = 0.52). The results of this meta-analysis indicated that both the levels of depressive symptoms in prenatally depressed women (p = 0.04) and non-depressed women (p < 0.00001) were statistically significantly lower in yoga groups than that in control groups.

**Figure 4:**
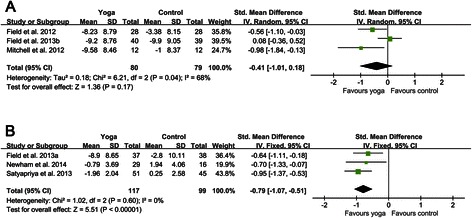
**Forest plots of effects of exercise-based yoga and integrated yoga on prenatal depression scores. (A)** Forest plot of the comparison of the exercise-based yoga group versus the control group for prenatal depression scores. **(B)** Forest plot of the comparison of the integrated yoga group versus the control group for prenatal depression scores.

As to yoga interventions, three RCTs adopted exercise-based yoga [53,54,56]; the remaining three RCTs adopted the integrated yoga [52,55,57]. Pooling trials according to the types of yoga interventions gave standardized mean difference of −0.41 (−1.01 to 0.18) for exercise-based yoga and −0.79 (−1.07 to −0.51) for integrated yoga (Figure 4). There was a certain degree of heterogeneity in the subgroup of exercise-based yoga (I^2^ = 68%, p = 0.04), while the heterogeneity in the subgroup of integrated yoga was not evident (I^2^ = 0, p = 0.60). The results of this meta-analysis revealed that the depression level of the exercise-based yoga group was not statistically significantly reduced (p = 0.17). However, the depression level of the integrated yoga group was significantly decreased (p < 0.00001).

## Discussion

Using an exhaustive and comprehensive search strategy, we identified six moderate quality RCTs to evaluate the intervention of yoga for prenatal depression. This study demonstrates that yoga can be helpful for pregnant women to alleviate symptoms of depression. Analysis of overall effect indicated that yoga intervention significantly reduced the level of maternal depression before parturition. Besides, the control groups of some included studies received some forms of social support or massage rather than usual care. Since these interventions are not belonging to simple usual care, the true effect of yoga may be underestimated.

In order to figure out the potentially diverse effects of yoga, these studies were divided by the types of participants and yoga interventions. A subgroup analysis of depressed and non-depressed women showed that both types apparently benefited from yoga treatment for antenatal depression. Results of the other subgroup revealed that integrated yoga intervention significantly reduced the level of prenatal depression, but the exercise-based yoga did not. The SMDs with 95% CIs were −0.79 (−1.07, −0.51) and −0.41(−1.01, 0.18), respectively. As a consequence, compared with exercise-based yoga, the integrated yoga may be a better choice for pregnant women.

Previous meta-analysis has found the limited-to-moderate evidence for short-term improvements of depression and anxiety in severity [58], but there are not any reports about prenatal depression. Another meta-analysis focused on exercise for antenatal depression, and showed a significant reduction in depression scores (SMD −0.46, 95% CI −0.87 to −0.05, p = 0.03, I^2^ = 68%) for exercise intervention relative to the comparison group [38]. However, Satyapriya et al. [57] reported that the depression level of yoga intervention group was significantly lower than that in the exercise group. Therefore, yoga seems useful to alleviate prenatal depression, and the results show that integrated yoga may be even more effective.

This meta-analysis included six moderate quality RCTs. Participants were recruited at their first or second ultrasound assessment (about 20 weeks gestation), including Hispanic, African-American, white and a few other races in the United States, India and the United Kingdom. The inclusion criteria and exclusion criteria of these RCTs were similar. All of the depressed participants met diagnostic criteria for depression on SCID. Most of the RCTs used the same rating scales to measure depression (CES-D). The program lengths of yoga and control groups of the six RCTs were about 12 weeks.

This study also has several limitations. First of all, since the baselines of each study were not exactly the same and the methods to generate random sequence were not clearly clarified in most trials, the selection biases cannot be controlled efficiently. Also, most RCTs used CES-D to measure the levels of depression, while one RCT used HADS [57] and another used EPDS [52]. The variable methods of measuring and reporting depression may contribute to a certain degree of heterogeneity. Using CES-D and SCID to assess pregnant women’s depression is not the best choice due to misinterpretation of somatic symptoms of pregnancy for certain items (e.g., tiredness, lack of energy). Instead, using Edinburgh Postnatal Depression Scale (EPDS) will be better. Another potential drawback involves the effect of study dropouts on the validity of study findings. In addition, trials with negative results are less likely to be published and more likely to be excluded from systematic review, which induces the literature towards positive findings. Finally, this study included six trials and 375 pregnant women. The small sample size may possibly lead to false positive conclusion.

More attention should be paid to use the standardized yoga as an intervention in future research, which may contribute to find out the best way to prevent and treat prenatal depression.

## Conclusions

With the limitations in mind, this article allows us to draw several conclusions regarding yoga for prenatal depression. Firstly, prenatal yoga may be helpful to decrease maternal depressive symptoms. Secondly, both the depressed and non-depressed pregnant women can benefit from yoga. Lastly, the integrated yoga seems more effective in treating depression than physical-exercise-based yoga.

## Additional files

Additional file 1: Table S1. Characteristics of 6 studies included in the meta-analysis. CES-D, Center for Epidemiological Studies Depression Scale; Structured Clinical Interview for DSM-IV; IVF, In Vitro Fertilization; IUGR, Intrauterine Growth Retardation; TAU, treatment-as-usual; POMS, Profile Of Mood States; HADS, Hospital Anxiety Depression Scale; EPDS, Edinburgh Postnatal Depression Scale.
Additional file 2: Table S2. Quality assessment of the studies included.
