# Supplementary material for: Yoga for prenatal depression: a systematic review and meta-analysis
Source: BMC Psychiatry. 2015 Feb 5;15:14. doi: 10.1186/s12888-015-0393-1 (PMC4323231; doi:10.1186/s12888-015-0393-1)
Supplement: Additional file 1: Table S1. — Characteristics of 6 studies included in the meta-analysis. CES-D, Center for Epidemiological Studies Depression Scale; Structured Clinical Interview for DSM-IV; IVF, In Vitro Fertilization; IUGR, Intrauterine Growth Retardation; TAU, treatment-as-usual; POMS, Profile Of Mood States; HADS, Hospital Anxiety Depression Scale; EPDS, Edinburgh Postnatal Depression Scale. [file 12888_2015_393_MOESM1_ESM.pdf]

Table 1 Characteristics of 6 studies included in the meta-analysis.

Article (publication year)	Country	Participants (N, diagnosis, age, gestation, cost, percentage of primiparous women in each group)	Exclusion	Intervention groups (program length, frequency, duration, practice mode)		Outcome measures	Results
				Yoga	Control		
Field et al. (2012)	United States	N=84, 18-40 years old, 18-22 weeks gestation, depressed (CES-D, SCID)	Medical illness, 40 years or older, other psychiatric condition, self-reported drug use or medications that might confound the depression effects	12 weeks, 2 ×20 min/ week (postures), in groups (8 women at each session)	Massage and standard prenatal care	CES-D	Significant difference favoring yoga over control group
Mitchell et al. (2012)	United States	N=24, 18-37 years old, 20 weeks gestation, depressed (SCID), be paid $20 for each session	Pregnant with more than one child, medical illness, >40 years old, having a co-morbid mental health issue, using drugs (i.e., prescribed or illicit)	12 weeks, 2 ×20 min/week (postures), in groups	12 parenting education sessions	CES-D, depressed affect, positive affect, interpersonal distress	Significant difference favoring yoga over control group
Field et al. (2013a)	United States	N=92, 18-37 years old, at an average of 22 weeks gestation, depressed (SCID), be paid $20 for each session	Pregnant with more than one child, medical illness, >40 years old, using drugs	12 weeks, 1 ×20 min/week (yoga combined with tai chi), in groups	Social support group: 12 weeks, 1 ×20 min/week	CES-D	No significant group difference

Field et al. (2013b)	United States	N=92, 20-38 years old, 22 weeks gestation, depressed (SCID), be paid $20 for each session	Other psychiatric complications conditions, pregnancy medical illness, maternal age older than 40-years-old and younger than 20, illicit drug use	12 weeks, 1 × 20 min/week (postures), in groups	Social support group: 12 weeks, 1 ×20 min/week	POMS, CES-D	No significant group difference
Satyapriya et al. (2013)	India	N=96, 20-35 years old, 18-20 weeks gestation, normal pregnancies (not always depressed), 88% in yoga and 87% in control group	Associated medical problems (diabetes, hypertension, etc.), multiple pregnancy, IVF pregnancy, previous history of IUGR, maternal physical abnormalities, fetal abnormality on ultrasound scanning, previous exposure to yoga	Integrated yoga: 16 weeks, 1h/d, 15min/10min lectures, 10min/5min breathing exercises, 15min/10min asana postures, 10min/20min pranayama and meditation, 10min/15min deep relaxation, in groups for one month and at home for remaining time	Standard antenatal exercises: 15min/10min lectures, 10min/5min loosening exercises, 15min/10min antenatal exercises, 10min/20min slow walking, 10min/15min supine rest	HADS depression	Significant difference favoring yoga over control group
Newham et al. (2014)	United Kingdom	N=45, >18 years old, healthy women, the second or early third trimester of an uncomplicated, singleton first pregnancy, 100% in both yoga and control group	With a medical illness, taking prescription medication, already practicing antenatal yoga	Integrated yoga: 8 weeks, mild, hatha form of yoga, including exercises, postures, and relaxation/breathing techniques, in groups (10-11 women/group)	TAU: 8 weeks	EPDS	Significant difference favoring yoga over control group

CES-D, Center for Epidemiological Studies Depression Scale; SCID, Structured Clinical Interview for DSM-IV; IVF, In Vitro Fertilization; IUGR, Intrauterine Growth Retardation; TAU, treatment-as-usual; POMS, Profile Of Mood States; HADS, Hospital Anxiety Depression Scale; EPDS, Edinburgh Postnatal Depression Scale.
