# Supplementary material for: Yoga for prenatal depression: a systematic review and meta-analysis
Source: BMC Psychiatry. 2015 Feb 5;15:14. doi: 10.1186/s12888-015-0393-1 (PMC4323231; doi:10.1186/s12888-015-0393-1)
Supplement: Additional file 2: Table S2. — Quality assessment of the studies included. [file 12888_2015_393_MOESM2_ESM.pdf]

Table 2 Quality assessment of the studies included.

Article	Eligibility criteria specified	Randomization conducted	Allocation concealment	Lost to follow-up Yoga	Lost to follow-up Control	Intension-to-treat analysis	Adequate outcome assessor blinding	Score
Field et al. (2012)	Yes	Yes	Unclear	Unclear	Unclear	Unclear	Yes	B
Mitchell et al. (2012)	Yes	Yes	Unclear	Unclear	Unclear	Unclear	Unclear	B
Field et al. (2013a)	Yes	Yes	Unclear	9	8	Unclear	Unclear	B
Field et al. (2013b)	Yes	Yes	Unclear	6	7	Unclear	Yes	B
Satyapriya et al. (2013)	Yes	Yes	Unclear	2	7	Unclear	Yes	B
Newham et al. (2014)	Yes	Yes	Yes	2	6	Unclear	Unclear	B
